# Identification of Norway Spruce MYB-bHLH-WDR Transcription Factor Complex Members Linked to Regulation of the Flavonoid Pathway

**DOI:** 10.3389/fpls.2017.00305

**Published:** 2017-03-09

**Authors:** Miguel Nemesio-Gorriz, Peter B. Blair, Kerstin Dalman, Almuth Hammerbacher, Jenny Arnerup, Jan Stenlid, Shahid M. Mukhtar, Malin Elfstrand

**Affiliations:** ^1^Department of Forest Mycology and Plant Pathology, Swedish University of Agricultural SciencesUppsala, Sweden; ^2^Department of Biology, University of Alabama at BirminghamBirmingham, AL, USA; ^3^Department of Chemistry and Biotechnology, Swedish University of Agricultural SciencesUppsala, Sweden; ^4^Department of Microbiology, Forestry and Agricultural Biotechnology Institute, University of PretoriaPretoria, South Africa

**Keywords:** MYB, *Picea*, yeast two-hybrid, TT2, TT8, TTG1, flavonoid

## Abstract

Transcription factors (TFs) forming MYB-bHLH-WDR complexes are known to regulate the biosynthesis of specialized metabolites in angiosperms through an intricate network. These specialized metabolites participate in a wide range of biological processes including plant growth, development, reproduction as well as in plant immunity. Studying the regulation of their biosynthesis is thus essential. While MYB (TFs) have been previously shown to control specialized metabolism (SM) in gymnosperms, the identity of their partners, in particular bHLH or WDR members, has not yet been revealed. To gain knowledge about MYB-bHLH-WDR transcription factor complexes in gymnosperms and their regulation of SW, we identified two bHLH homologs of *At*TT8, six homologs of the MYB transcription factor *At*TT2 and one WDR ortholog of *At*TTG1 in Norway spruce. We investigated the expression levels of these genes in diverse tissues and upon treatments with various stimuli including methyl-salicylate, methyl-jasmonate, wounding or fungal inoculation. In addition, we also identified protein-protein interactions among different homologs of MYB, bHLH and WDR. Finally, we generated transgenic spruce cell lines overexpressing four of the Norway spruce *At*TT2 homologs and observed differential regulation of genes in the flavonoid pathway and flavonoid contents.

## Introduction

As trait complexity grows larger in organisms, gene families encoding regulatory proteins, such as transcription factors (TFs) are generally expanded and this expansion contributes to the evolutionary diversification of traits, e.g., developmental fate or specialized metabolism (SM) (Lang et al., 2010). The concerted action of multiple classes of TFs in regulatory protein complexes may allow for further trait diversification (Singh, 1998). A well-known example of a specific regulatory protein complex driving the diversification of multiple traits in plants is the R2R3-MYB/basic helix-loop-helix (bHLH)/WD-Repeat (MBW) complex (Pesch et al., 2015; Xu et al., 2015). The MBW members cooperatively participate to form a transcriptional activating complex in which the bHLH member plays a central role and interacts with both, the MYB transcription factor and the WD40 protein. Collectively, these three members are required for the regulation of specialized metabolic pathways and of specific developmental programs in higher plants (Baudry et al., 2004; Feller et al., 2011; Tominaga-Wada and Wada, 2014; Xu et al., 2015). The MBW complex is essentially modular in its organization in diverse plant systems (Xu et al., 2015) and is well studied in the model plant *Arabidopsis thaliana*. For instance, Arabidopsis WD40 protein is represented by the single-copy gene *Transparent Testa Glabra 1* (*TTG1*) that controls all traits associated with MBW complexes (Tominaga-Wada et al., 2011). Three bHLH proteins, TT8, EGL3 and GL3, act in a partially redundant manner in the *Arabidopsis*' MBW complex (Nesi et al., 2001; Heim et al., 2003; Toledo-Ortiz et al., 2003; Baudry et al., 2004; Pires and Dolan, 2010). Finally, the MYB TFs regulate the expression of specific genes involved in the biosynthesis of specialized metabolites (SM) (Dias et al., 2003; Baudry et al., 2004; Gonzalez, 2009). Angiosperms and gymnosperms diverged around 300 million years ago (Savard et al., 1994; Lu et al., 2014). The most widespread among the extant gymnosperms are the conifers, which dominate large forest areas, especially in boreal and montane forests in the Northern Hemisphere. Despite their evolutionary history and their ecological importance, our knowledge of the MBW complex in conifers is episodic and focused on R2R3-MYB TFs, which have been relatively well studied regarding their regulatory roles in the biosynthesis of different classes of SMs. Xue et al. (2003) identified 10 black spruce (*Picea mariana)* R2R3-MYBs. *Pm*MBF1, a member of R2R3-MYBs family, is capable of inducing pigment accumulation during transient overexpression in maize cell lines and caused transactivation of the anthocyanidin-related *Bz2* promoter in spruce and larch cell lines. 18 R2R3-MYBs have been discovered in white spruce (*Picea glauca*) and loblolly pine (*Pinus taeda*) (Bedon et al., 2007). Tissue specific expression patterns suggest potential roles of particular members of R2R3-MYBs in lignin biosynthesis. Indeed, the roles of at least two of the pine R2R3-MYB TFs in lignification was later confirmed as white spruce lines overexpressing these two pine R2R3-MYB TFs showed increased lignin accumulation in cell walls and induction of genes in the shikimate and monolignol biosynthetic pathways (Bomal et al., 2008). In addition, white spruce lines overexpressing the white spruce TF *Pg*MYB14 showed increased terpene and flavonoid accumulation (Bedon et al., 2010). Similar to *Arabidopsis*, there appears to be a high degree of complexity associated with the R2R3-MYB component of the MBW complex in conifers, resulting in specific transcriptional responses. It has been recently shown that a similar set of genes exhibit opposing transcriptional outputs in the transgenic lines overexpressing diverse members of the R2R3-MYB TF family (Bomal et al., 2008, 2014), suggesting distinct actions of closely related R2R3-MYB TFs in conifers. In two independent studies, Bedon et al. (2010) and Xue et al. (2003) identified bHLH-binding motifs in the upstream regulatory regions of R2R3-MYB TFs, suggesting bHLH-MYB regulatory interactions that carry out a downstream gene activation cascade. However, there are very few reports on potential coniferous bHLH or WD40 members of the MBW complex. In addition, there are no reports involving conifer *WD40* genes, while a stress-induced *bHLH* gene in Norway spruce (*Picea abies*) encoding a predicted protein with similarity to Arabidopsis TT8 was superficially reported by Lundén et al. (2015).

Given the previous reports of spruce transcripts with similarity to *Arabidopsis TT8* and *TT2* genes where two different genes have been identified as potential spruce *At*TT2 homologs (Xue et al., 2003; Arnerup et al., 2011; Lundén et al., 2015), the aim of this study was to survey and identify potential members of the MBW complex in Norway spruce (*Picea abies*). We hypothesized that the Norway spruce genome includes a complete MBW complex repertoire, including WD40 proteins homologous to TTG1. We also hypothesized that Norway spruce would harbor several potential *At*TT2 homologs in the R2R3-MYBs of subgroup S5 as seen in other species (Stracke et al., 2001; Yoshida et al., 2008; Schaart et al., 2013; Soler et al., 2015). Consistent with our hypotheses, one *AtTTG1* homolog, two *AtTT8* homologs and six putative *AtTT2* homologs were identified in the Norway spruce genome, including the previously described *TT2* (Arnerup et al., 2011). As we could identify two *TT8* and six *TT2* paralogs, we hypothesized that these sets of paralogs would display differentiation in protein function and/or expression patterns consistent with the theory of sub-functionalization (Innan and Kondrashov, 2010). Therefore, we quantified the expression patterns of the paralogs in different tissues and in response to various hormone analogs simulating abiotic and biotic stress and assessed their biophysical interaction with each other and the Norway spruce *At*TTG1 homolog in a Yeast-two-Hybrid (Y2H) assay. In angiosperms, members of the MBW complex are known to regulate late biosynthetic genes in the flavonoid biosynthetic pathway when overexpressed in combination or as individual genes (Baudry et al., 2004; Mellway et al., 2009; Matus et al., 2010; Schaart et al., 2013). Thus, in order to elucidate if the previously identified spruce *At*TT2 homologs (Xue et al., 2003; Arnerup et al., 2011) control secondary metabolism, we created transgenic Norway spruce cell lines overexpressing these two R2R3-MYB TFs and two of the newly identified R2R3-MYB TFs that showed transcriptional activity. We measured the expression levels of genes in the flavonoid biosynthetic pathway as well as the flavonoid, neolignan and stilbene contents of the transgenic lines.

## Materials and methods

### Plant material

Four-year-old Norway spruce (*Picea abies* [L.] *Karst*.) genotypes from the Forestry Research Institute of Sweden were used for tissue dissection and RNA extraction. The plants were placed at 25°C and 16 h photoperiod in a growth chamber for 3 weeks before sampling. Tissues were obtained from roots and twigs from the plants. The phloem and bark were separated with a knife from the sapwood in both, twigs and roots, and shoots were isolated from the tip of the twigs. In total, five samples were taken per plant. Each of the four genotypes was used as one independent biological replicate. All tissues were frozen separately in liquid nitrogen and stored at −80°C until further use.

The wild type Norway spruce embryogenic cell line 95:61:21 (Högberg et al., 1998) was used for measuring expression levels of candidate genes under abiotic stress conditions, for *Agrobacterium*-mediated cell transformation and for chemical analysis.

The plant material in Supplemental Material 8 is described in Arnerup et al. (2013).

### *In vitro* treatments

Norway spruce cells (line 95:61:21), grown on half-strenght LP agar (von Arnold and Eriksson, 1981) without plant growth regulators, were treated with several types of abiotic stress for 48 h at 21°C. The abscisic acid treatment was based on the addition of 8 μg/ml of abscisic acid to the medium. To study the effect of jasmonic acid and salicylic acid in gene expression (Arnerup et al., 2013), unsealed plates with cells were placed in a sealed jar and a cotton ball with 25 μL of either, 10% methyl salicylate or 10% methyl jasmonate, was placed inside of the jars next to the plates in the beginning of the treatment and after 24 h. At harvest, cells were collected, frozen in liquid nitrogen and stored at −80°C until further use.

### Norway spruce transformation and line selection

Transformation of the WT cell line 95:61:21 was carried out according to Minina et al. (2013). Transgenic calli were picked from the plates after 3 weeks. Transgenic lines were selected and verified by PCR and the expression levels of the transgenes were assessed by qPCR.

For quantification of target genes, three Norway spruce transformant lines per construct overexpressing their transgene between 10 and 20 times more than the control lines were selected (Supplemental Material 1). Expression of the Norway spruce early biosynthetic genes *PAL1* (Koutaniemi et al., 2007) and *CHS* (Richard et al., 2000), and the late biosynthetic genes *LAR1, LAR2, LAR3, LAR4, ANR2, ANR3*, and *ANR5* (Arnerup, 2011; Hammerbacher et al., 2014) of the flavonoid biosynthetic pathway were tested in these lines.

### RNA extraction and cDNA synthesis

For lignified tissues, total RNA extraction was done essentially according to the protocol by Chang et al. (1993) with modifications described in Arnerup et al. (2011). RNA extraction of *in vitro* material was performed using the RNeasy Plant Mini Kit (Qiagen) following the manufacturer's instructions. Purified RNA samples were treated with DNase1 (Sigma Aldrich) according to the manufacturer's instructions and RNA concentration was determined with the NanoDrop (Spectrophotometer ND 1000, Saveen Werner). Five hundred ng of total RNA were reverse transcribed to cDNA with the iScript™ cDNA Synthesis Kit (Bio-Rad) in a total reaction volume of 20 μl according to the manufacturer's instructions.

### Candidate identification and primer design

To identify candidates, amino acid sequences of TT2, TT8, and TTG1 were used to query the Norway spruce genome version 1.0 (www.congenie.org) and GenBank (http://www.ncbi.nlm.nih.gov/genbank/) using Blastp.

For cloning the different candidates, Primer3 (http://biotools.umassmed.edu/bioapps/primer3_www.cgi) was used to predict and design PCR primers (20 nucleotide length and Tm 60°C) covering the whole predicted ORF of the transcripts (gene models can be found in Supplemental Material 7). Primer quality and properties were checked at www.bioinformatics.org/sms2/pcr_primer_stats and AttB borders were added to the sequences before primers were synthesized at TAG Copenhagen (Supplemental Material 1).

For quantitative RT-PCR, Primer3 was used to design primers amplifying sequences of 120–150 bp within the predicted ORF of the transcript sequences and primer quality and properties were also checked at www.bioinformatics.org/sms2/pcr_primer_stats before primers were synthesized at TAG Copenhagen (Supplemental Material 2).

### Candidate isolation and vector construction

For the isolation of candidate genes, primer pairs with AttB borders specific for each of the candidates were used (Supplemental Material 2). A 50 μl PCR-mixes consisting of 1x Dream-Taq green buffer (Thermo Fischer Scientific), 0.2 μM of each of the primers, 0.2 mM dNTPs (Qiagen), 1.25 U Dream-Taq Polymerase (Thermo Fischer Scientific), a final concentration of MgCl_2_ of 3.25 mM, and 0.5–5 ng/μl reaction volume of Norway spruce cDNA, was prepared. The PCR conditions were as follows: initial denaturation at 95°C for 5 min, followed by 35 cycles of: 30 s at 95°C, 30 s at 57°C, and 2 min at 72°C and a final elongation step of 7 min at 72°C.

The PCR products were cloned into the Gateway pDONR/Zeo (Thermo Fisher Scientific) entry vector according to manufacturer's instructions. Entry vectors were purified using the PlasmidMini Kit® (Qiagen) and 500 ng were sent to Macrogen (Amsterdam, The Netherlands) for Sanger sequencing and the sequences were used to enable phylogenetic analyses.

To prepare the vectors for transformation of Norway spruce the isolated genes were transferred to the pMDC32 (Curtis and Grossniklaus, 2003) vector by LR recombination. The resulting vectors was verified by test-digestion and sequencing.

To prepare the destination vectors for the yeast two-hybrid experiment, LR recombination reactions (Thermo Fisher Scientific) were used to transfer our isolated genes into pDest-AD-CYH2 and pDest-DB destination vectors (Mukhtar et al., 2011) according to the manufacturer's instructions. Vectors were purified using the PlasmidPrep minikit® (Qiagen) and an aliquot of each vector was sent for Sanger sequencing to Macrogen (Amsterdam, The Netherlands).

### Phylogenetic analyses

Predicted amino acid sequences were obtained by exporting DNA sequences of the isolated genes to ORF finder (www.ncbi.nlm.nih.gov/projects/gorf/). Sequences were translated and amino acid sequences were imported into MEGA6 (Tamura et al., 2013) and aligned by ClustalW algorithm with default settings (Gap opening penalty 15, gap extension penalty 6.66, IUB DNA weight matrix and transition weight 0.5).

Due to the presence of a single member representing *TTG1* in Norway spruce and *Arabidopsis*, the construction of a phylogenetic tree was not possible and we used the LALIGN analysis (http://www.ch.embnet.org/software/LALIGN_form.html) to calculate the identity and similarity between the protein sequences of our Norway spruce candidate, *Pa*WD40-1, and *At*TTG1.

**Figure 1:**
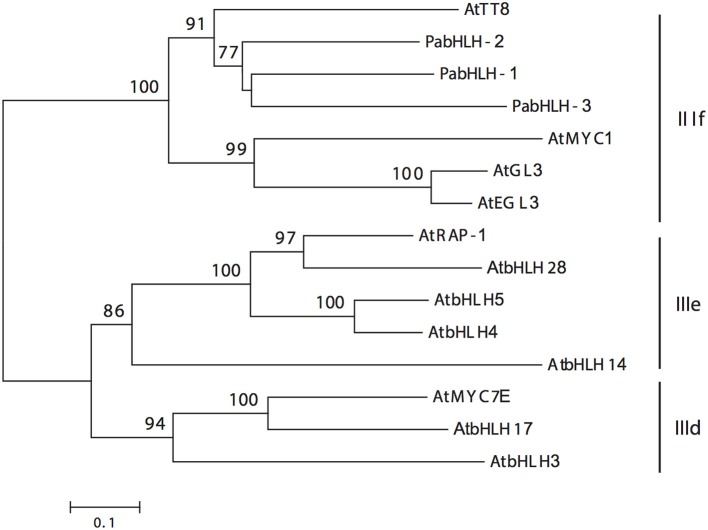
**Unrooted neighbor-joining tree of the three Norway spruce bHLH proteins and the ***Arabidopsis thaliana*** bHLH proteins of subgroups IIId, IIIe, and IIIf (Pires and Dolan, 2010), which are indicated by the black vertical lines**. The numbers on the braches correspond to the bootstrap support (1000 replications).

**Figure 2:**
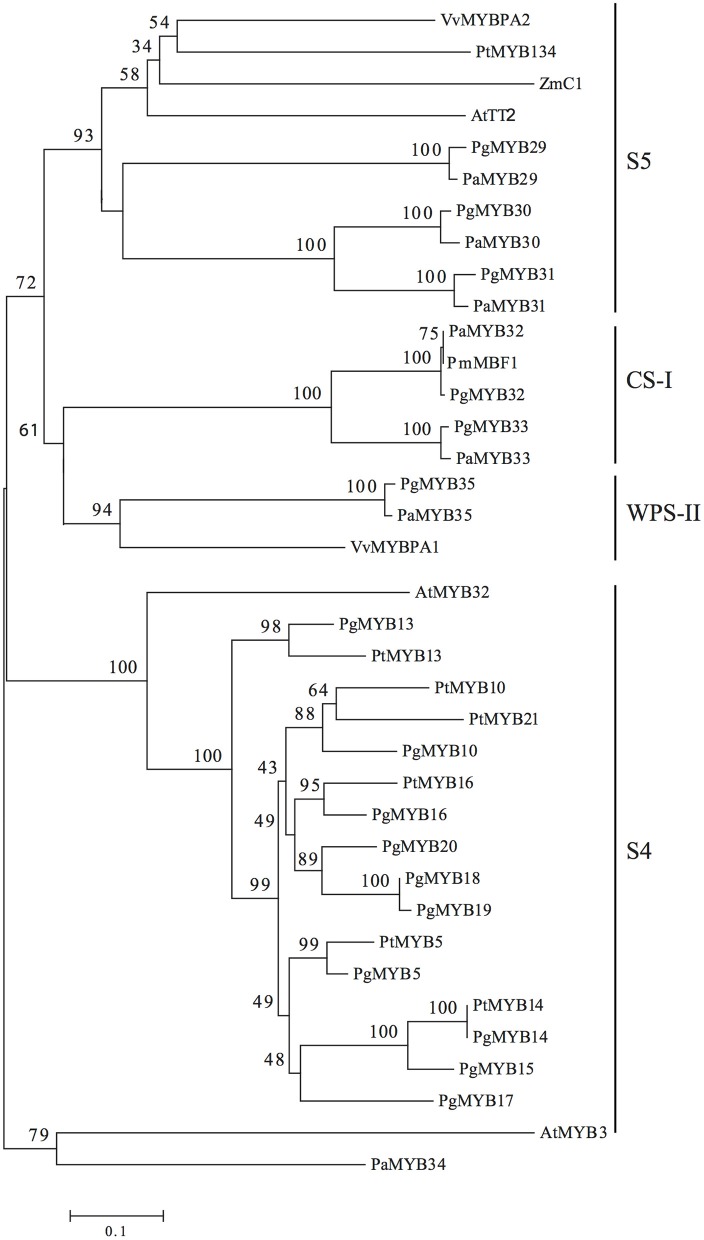
**Unrooted neighbor-joining tree of R2R3-MYB subgroups 4 and 5 in Pinaceae**. Angiosperm R2R3-MYB proteins are included as a reference. Black lines indicate subdivisions within the tree. Pg, *Picea glauca*; Pm, *Picea mariana*; Pa, *Picea abies*; At, *Arabidopsis thaliana*; Vv, *Vitis vinifera*; Zm, *Zea mays*; Pt, *Populus tremuloides*. The numbers on the braches correspond to the bootstrap support (1000 replications).

EMBL/GenBank accession numbers for Figures 1, 2 can be found in Supplemental Material 3.

### Quantitative RT-PCR

For preparation of standards for the qPCR reaction, PCR reactions consisted of 1x Dream-Taq green buffer (Thermo Fischer Scientific), 0.25 μM of each of the qPCR primers, 0.2 mM dNTPs (Qiagen), 6.25 U Dream-Taq Polymerase (Thermo Fischer Scientific) and 1 μl of Norway spruce cDNA. Initial denaturation was at 95°C for 5 min, followed by 35 cycles of: 15 s at 95°C, 20 s at 60°C, and 20 s at 72°C and a final elongation step of 3 min at 72°C. PCR products were cloned into TOPO®-TA vectors (Thermo Fischer Scientific) following the manufacturer's instructions. Plasmids were purified using The Qiagen PlasmidMini Kit® and dilution series were then prepared from 10^8^ to 10^3^ copies/μl.

Quantitative PCR reactions were performed with the SsoFast™ EvaGreen® Supermix (Bio-Rad) according to the instructions in the manual, using 0.3 μM of each primer and 1 μl of Norway spruce cDNA. The qPCRs were carried out in an iQ5™ Multicolor Real-Time PCR Detection System thermo cycler (Bio-Rad) using a program with a 30 s initial denaturation step at 95°C, followed by 40 cycles of 5 s denaturation at 95°C and 10 s at 60°C. Melt curve analyses were used to validate the amplicon. Two repetitions per standard, sample, and negative control were run.

For the tissue panel, 2 ΔΔCT values and standard curves were used to calculate expression levels. *Elongation Factor 4 Alpha* (*EF4*α) (Vestman et al., 2011) was used as a reference gene to normalize the values of the target genes in each sample. For each gene, relative expression levels were calculated by comparing the expression levels in a tissue to the average in all the tissues that were analyzed.

For the Stress panel and the transgenic cell lines, Ct values were imported into REST (Pfaffl et al., 2002) software 2009 and *EF4a* was used as a control gene. Expression levels of treated and transgenic cells were compared to untreated and untransformed cells, respectively.

### Yeast two-hybrid assay

DB and AD plasmids were individually transformed into haploid yeast (*S. cerevisiae*) strains Y8930 (MATα) and Y8800 (MATa) to create baits and preys, respectively as described by Mukhtar et al. (2011). Briefly, Y8930 and Y8800 strains were grown in liquid YEPD overnight as pre-cultures. Cultures were prepared with a starting OD of 0.1 and the cells were harvested and prepared for transformations, once they reached an OD between 0.4 and 0.6. The baits and preys were selected on Difco™ yeast nitrogen base (YNB) with leucine dropout (-L) and tryptophan dropout (-T) selective media, respectively. The haploid bait and prey yeast strains were pairwise mated overnight in YEPD. The diploid yeast cells were selected onto YNB -LT selective liquid media, and subsequently spotted onto YNB -LTH as well as -LH containing cycloheximide (CHX) selective media. In addition, we also determined the strength of protein-protein interaction by supplementing–LTH and -LH media with 3-Amino- 1, 2, 4-trizole (3AT), a competitive inhibitor of histidine biosynthesis. Yeast growth on–LTH but not on -LH containing CHX media were scored as positive interactions. Yeast growth found on both –LTH and –LH containing CHX were due to *de* novo autoactivation and hence removed from the data set.

### Analysis of phenolic specialized metabolites in overexpression lines

Norway spruce cells from suspension cultures of three transformant lines per construct were harvested, stored immediately in liquid nitrogen, and later freeze-dried. The weights of the dried samples were annotated and the samples were then ground in a shaking ball mill using metal beads. Chemical content was measured using the same method as described by Wadke et al. (2016) and Hammerbacher et al. (2014).

Variances in the chemical contents of specific compound groups were analyzed by Principal Component Analysis (PCA, Past 3; Hammer et al., 2001). A one-way ANOVA with Dunns Post-test (GraphPad Prism 5.0) was used to detect differences in specific compounds between lines.

## Results

### Identification of homologs of *At*TT8, *At*TTG1, and *At*TT2 in norway spruce

Three putative Norway spruce bHLH genes, *PabHLH-1* (2019 bp), *PabHLH-2* (2274 bp), and *PabHLH-3* (1071 bp), encoding TFs homologous to *At*TT8 were isolated and cloned from Norway spruce. In all three cases, the sequenced ORFs matched the length of the high-confidence gene models in the Norway spruce genome assembly (Nystedt et al., 2013). The predicted amino acid sequences clustered together with *At*TT8 in a well-supported clade (Figure 1) in subgroup IIIf, identified by Pires and Dolan (2010). *Pa*bHLH-1/2 both possess the characteristic 30 AA domain 8 (Pires and Dolan, 2010) sequence N-terminally in the amino acid sequence, while this domain appears to be truncated in *Pa*bHLH-3. Furthermore, the predicted *Pa*bHLH3 appears to lack the conserved bHLH domain (Supplemental Material 4) and for this reason it was not used in further experiments.

Based on the high-confidence gene model with a predicted protein homologous to TTG1 in the Norway spruce genome v.1.0 (Supplemental Material 5), the cDNA sequence of *PaWD40-1* was isolated. The sequence covered the ORF gene (1020 bp) and was identical to the gene model predicted in the Norway spruce genome assembly. Alignment of the amino acid sequences of the *Arabidopsis* TTG1 (341 aa) and our candidate, PaWD40-1 (339 aa), showed a highly conserved structure between the two proteins (Supplemental Material 5), 85% similarity and 66% identity, suggesting that *PaWD40-1* is the Norway spruce ortholog of *AtTTG1*.

Seven transcripts encoding R2R3-MYB TFs homologous to *At*TT2, the only member of the R2R3-MYB subgroup 5 in *Arabidopsis* (Stracke et al., 2001), were identified in Norway spruce. To achieve this, we used the amino acid sequence of *At*TT2 to query the Norway spruce genome 1.0 (Nystedt et al., 2013). Seven gene models were identified (Supplemental Material 5) covering the full ORF except for the one corresponding to *PaMYB29*, which lacked the 3′ end of the ORF based on the previously isolated GenBank accession JF810440. We successfully isolated the full ORF sequences for the seven candidates: *PaMYB29* (909 bp), *PaMYB30* (1116 bp), *PaMYB31* (1074 bp), *PaMYB32* (1149 bp), *PaMYB33* (1041 bp), *PaMYB34* (1077 bp), and *PaMYB35* (1173 bp). All seven genes were named following the numeration established for the already published white spruce R2R3-MYB TFs (Bedon et al., 2007, 2010; Duval et al., 2014). All isolated R2R3-MYB TFs showed conserved R2R3 domains, located in the N-terminal site of the predicted protein, and a highly variable C-terminal sequence (Supplemental Material 6).

Phylogenetic analyses of the amino acid sequences revealed three subdivisions within the subgroup, which we classified as S5, WPS-II and CS-I based on the MYB classification presented by Soler et al. (2015) (Figure 2). Subgroup S5 enclosed the TFs with the highest homology to *At*TT2 and its orthologs from poplar (*Pt*MYB134), grape (*Vv*MYBPA2) and maize (C1) into a single clade with high bootstrap support. Orthologus sequences from Norway spruce, represented by *Pa*MYB29, which was previously named *Pa*TT2 (Arnerup, 2011), together with its paralogs *Pa*MYB30 and *Pa*MYB31, grouped closely together with their respective ortholog from white spruce in a well-supported group corresponding to the previously identified subgroup 5. A second subgroup previously identified as WPS-II (Soler et al., 2015), only included *Pa*MYB35, the Norway spruce ortholog of *Vv*MYBPA1 (Bogs et al., 2007), which regulates proanthocyanidin biosynthesis in grapevine. Finally, we identified subgroup CS-I (Conifer-specific I), which comprises*Pa*MYB33 and *Pa*MYB32, the Norway spruce homolog of the *Picea mariana* MYB TF MBF1 (Xue et al., 2003). *PaMYB34* has no known homologs in any other species and could not be placed phylogenetically in any of the MYB subgroups 4 or 5, therefore, it was not included in any further analyses. The pair of closely related paralogs *Pa*MYB30/*Pa*MYB31 showed high levels of identity (66.7%) and similarity (80.4%) over their whole protein sequences, respectively (Supplemental Material 6). The BLAST analysis against the conifer genomes and GenBank showed that the white spruce genome contains orthologous scaffolds for all the genes, and the loblolly pine genome contains orthologous scaffolds for five, including the two members of subgroup CS-I. GenBank sequences homologous to our candidates were also found in other conifers (Supplemental Material 7). No MYB proteins homologous to the subgroup CS-I (*Pa*MYB32 and *Pa*MYB33) members could be found outside the *Pinaceae* family.

### Differential physical interaction patterns of MYB subgroup 5 transcription factors in norway spruce

**Figure 3:**
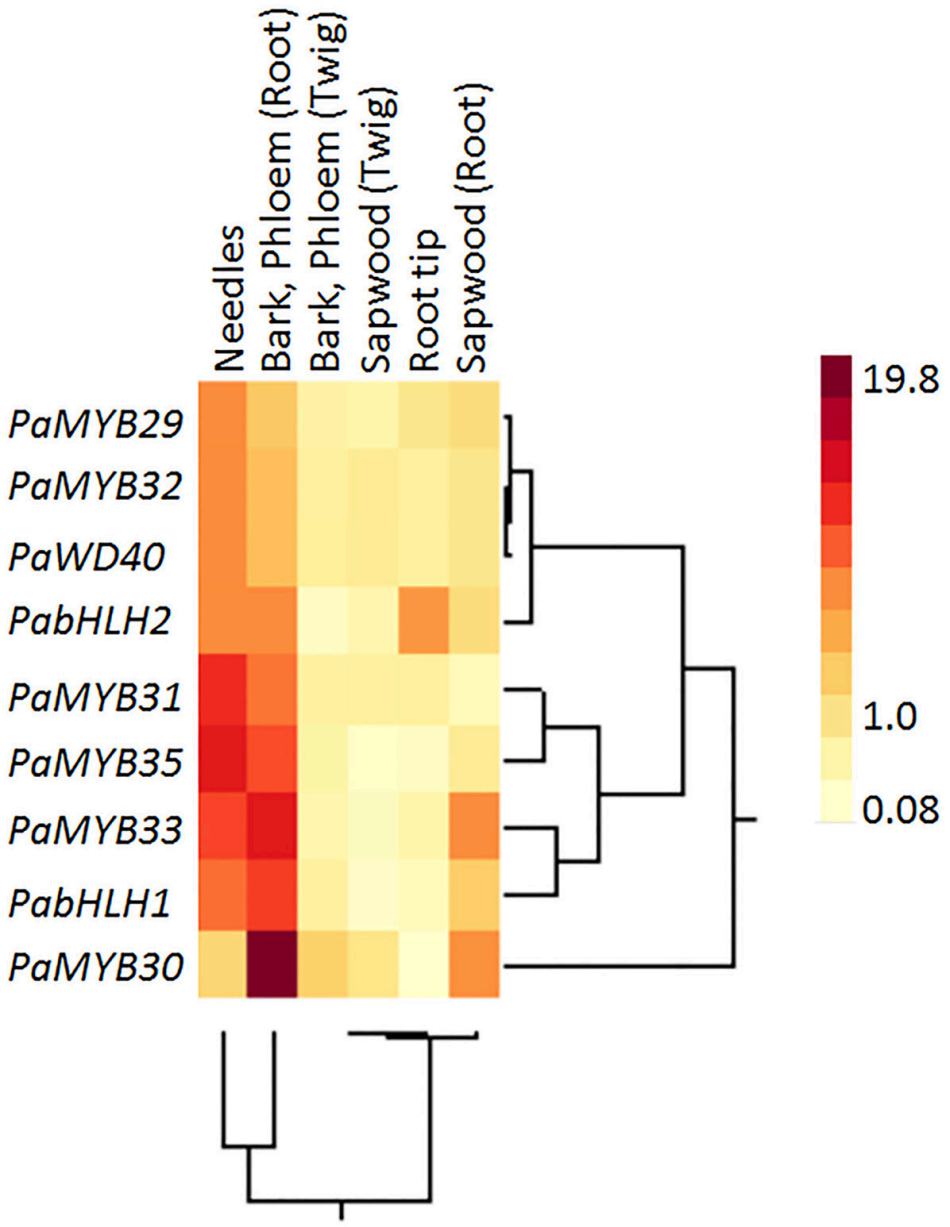
**Two-way cluster of the relative expression of ***PaWD40***, ***PabHLH*** and ***R2R3 MYB*** genes in specific Norway spruce tissues measured by qPCR**. The expression in a specific tissue compared to the average expression over all tissues. Four independent Norway spruce genotypes were used for this experiment.

**Figure 4:**
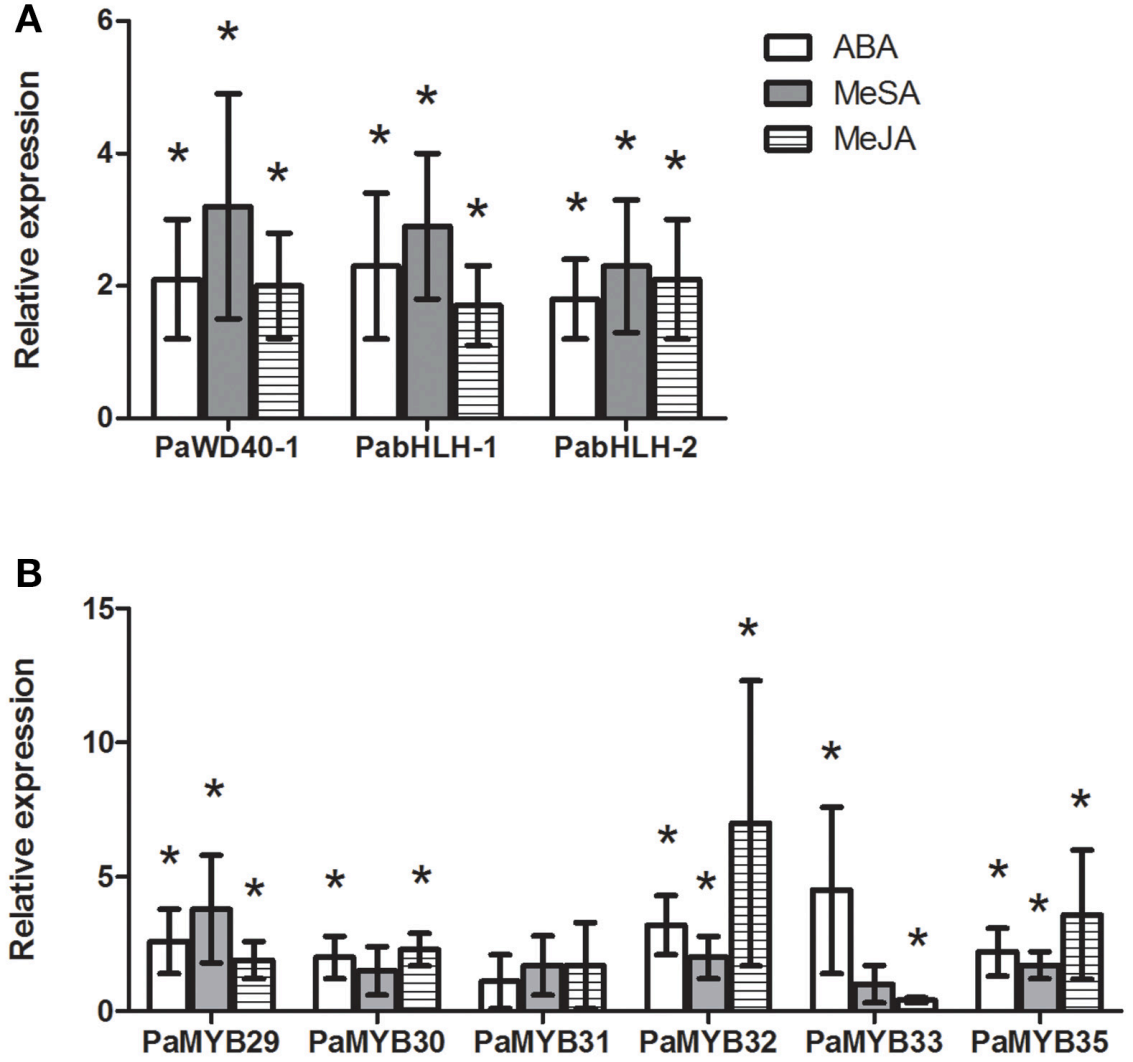
**Relative expression in Norway spruce of ***PaWD40-1, PabHLH-1, PabHLH-2*** (A)** and six *PaMYB* genes **(B)** in Norway spruce cell lines treated with ABA (open bars), MeSA (shaded bars) or MeJA (hashed bars) compared to untreated controls (*n* = 3). An asterisk (^*^) indicates significant regulation of the gene (*p* < 0.05, *T*-test) relative to the untreated control.

**Figure 5:**
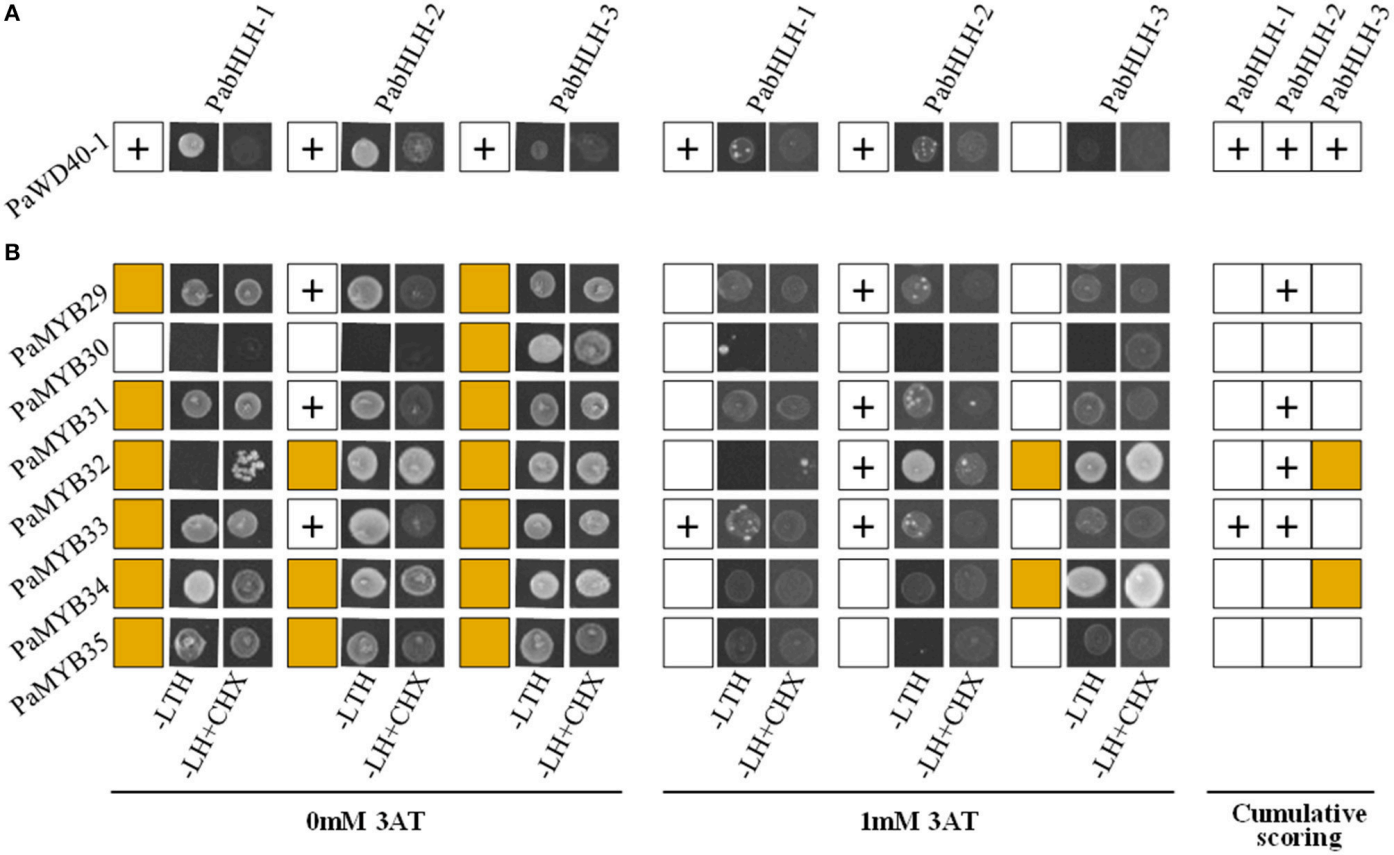
**Interaction of ***P. abies*** proteins using yeast-two-hybrid (Y2H). (A)** Interaction between PaWD40-1 and bHLH proteins. **(B)** Interaction between bHLH and MYB transcription factors. Plus sign (+) indicates positive interaction between a protein pair tested at 0 mM 3-Amino-1,2,4-triazole (3AT) or 1 mM 3AT. Brown box indicates autoactivations at both 0 mM 3AT or 1 mM 3AT conditions. Cumulative scoring encompasses positive interactions at any given conditions.

We did not observe any significant difference between the expression patterns of *PabHLH-1* and *PabHLH-2* in different tissues (Figure 3) and upon treatments with diverse hormones (Figure 4). Furthermore, both proteins interacted with *Pa*WD40-1, when *Pa*WD40-1 was expressed as the bait in the yeast two-hybrid assay (Figure 5, Supplemental Material 10). However, our yeast two-hybrid experiment revealed differences in interaction patterns between our bHLH and R2R3-MYB candidates. Specifically, we showed direct physical interactions of *Pa*bHLH-2 expressed as bait with *Pa*MYB29, PaMYB31, and *Pa*MYB33 and with *Pa*MYB32 when expressed as prey. *Pa*bHLH-1, on the other hand, only interacted with *Pa*MYB33 when expressed as bait (Figure 5, Supplemental Material 10). This suggests divergent roles of *Pa*bHLH-1 and *Pa*bHLH-2 in conjunction with MYB TFs in Norway spruce.

### Specific expression patterns of norway spruce MYBs

In contrast to the *bHLH* genes, the expression analysis showed significant differences in tissue specificity and hormone inducibility between our MYB genes (Figures 3, 4). Some tissues like shoots, phloem and root bark were considerably more transcriptionally active than sapwood, where only *PaMYB29, PaMYB32* and *PaMYB33* showed significant expression levels (Figure 3). Furthermore, we detected the transcripts of *PaMYB29* and *PaMYB32* in almost all tissues analyzed. Being these two transcripts the most ubiquitous and active, we analyzed the expression of *PaMYB29* and *PaMYB32* and a set of flavonoid biosynthesis genes in the phloem of 4-year old Norway spruce plants wounded or inoculated with *H. parviporum*. *PaMYB29, PaPAL1*, and some of the genes involved in the later steps of flavonoid biosynthesis that were tested showed induction after wounding and inoculation, while *PaMYB32* was neither induced by wounding or inoculation after 3 days, and only showed a small, but significant upregulation 7 days after inoculation (Supplemental Material 8).

The identified R2R3-MYB genes responded to hormonal treatments except for *PaMYB31*, which was expressed at very low levels. For the other MYB genes, our data showed that they responded to ABA and MeJA treatments, while MeSA treatment caused no or low induction for all MYB genes except for *PaMYB29* (Figure 4B). Interestingly, the two closely related paralogs, *PaMYB32* and *PaMYB33*, showed opposite transcriptional responses to treatment with MeJA (Figure 4B). While *PaMYB32* was strongly upregulated, *PaMYB33* was repressed. The expression analysis revealed that the highly expressed *PaMYB29* and *PaMYB32* genes were further induced by all treatments (Figure 4B).

### Norway spruce R2R3-MYB TFs of the subgroup 5 differentially regulate genes involved in the flavonoid biosynthesis pathway

To elucidate if *Pa*MYB29 and *Pa*MYB32, the previously identified (Xue et al., 2003; Arnerup et al., 2011) and also most highly expressed spruce *At*TT2 homologs, control secondary metabolism in Norway spruce we created transgenic cell lines overexpressing these two genes and two of the newly identified R2R3-MYB genes. These were the *PaMYB32* paralog, *PaMYB33*, and *PaMYB35*, the only representative of the flavonoid-related subgroup WPS-II in the Norway spruce genome. Twenty independent hygromycin-resistant Norway spruce cell lines were isolated 3 weeks after *Agrobacterium*-mediated transformation for each construct. The presence of the transgene (pMDC32::PaMYB29, pMDC32::PaMYB32, pMDC32::PaMYB33, or pMDC32::PaMYB35) was verified by PCR and the relative expression levels of the MYBs were determined by qPCR. Three lines per construct overexpressing the targeted MYB TF 10 to 20 times were selected for subsequent analyses together with the WT line.

**Figure 6:**
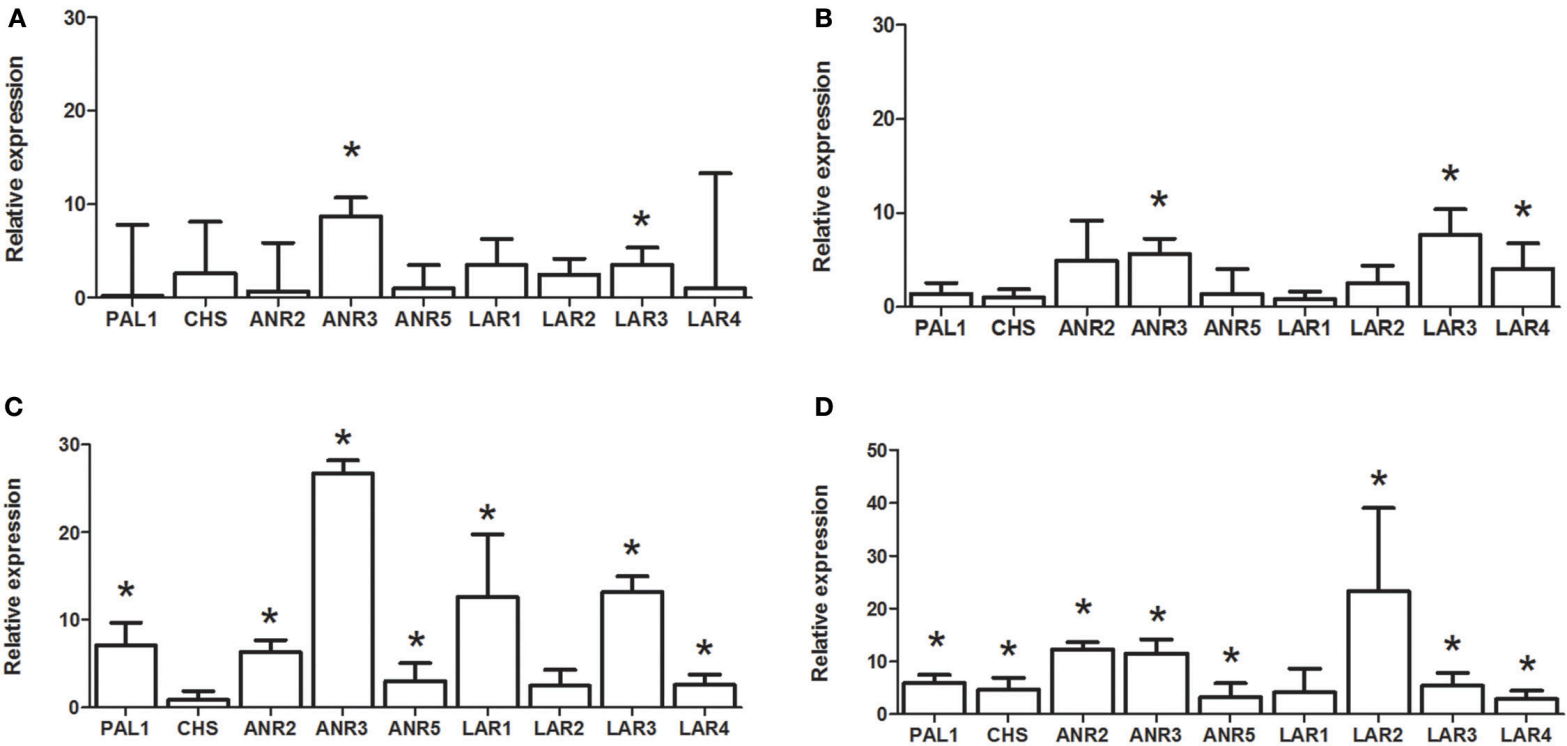
**Expression of genes in the flavonoid biosynthetic pathway in ***PaMYB*** overexpressing lines; OE-***PaMYB29*** (A)**; OE-*PaMYB32* **(B)**; OE-*PaMYB33* **(C)** and OE-*PaMYB35* **(D)** measured by qPCR. The graphs show average relative gene expression of three independent over expressing cell lines per construct compared to the untransformed WT line. An asterisk (^*^) indicates significant regulation of the gene (*p* < 0.05, *T*-test) relative to the untransformed WT line.

We measured expression levels of *PaPAL1, PaCHS* and eight genes in the late flavonoid biosynthetic pathway (LBGs) in Norway spruce transgenic cells lines overexpressing *PaMYB29, PaMYB32, PaMYB33* or *PaMYB35* (Figure 6). Our results showed that overexpression of *PaMYB33* and *PaMYB35* induced expression of the early biosynthetic genes in the flavonoid pathway *PaPAL1* and *PaCHS*, while *PaMYB29* and *PaMYB32* did not induce these genes. Overexpression of all four R2R3-MYB TFs upregulated the expression of late biosynthetic genes. Our results suggested, however, that the four R2R3-MYB genes target different genes downstream in the flavonoid pathway. *PaMYB29* overexpression induced *PaANR3* and *PaLAR3*, while *PaMYB32* overexpression upregulated *PaANR3, PaLAR3*, and *PaLAR4*. Overexpression of both *PaMYB33* and *PaMYB35* induced the expression of all the LBGs that we tested (*PaANR2, PaANR3, PaANR5, PaLAR1, PaLAR2, PaLAR3*, and *PaLAR4*) except for specific members of the *PaLAR* gene family (*PaLAR2* in the case of *PaMYB33* and *PaLAR1* in the case of *PaMYB35*) (Figure 6). In all cases, the induction of transcription by *PaMYB33* and *PaMYB35* overexpression was higher than the induction by *PaMYB29* and *PaMYB32*.

**Figure 7:**
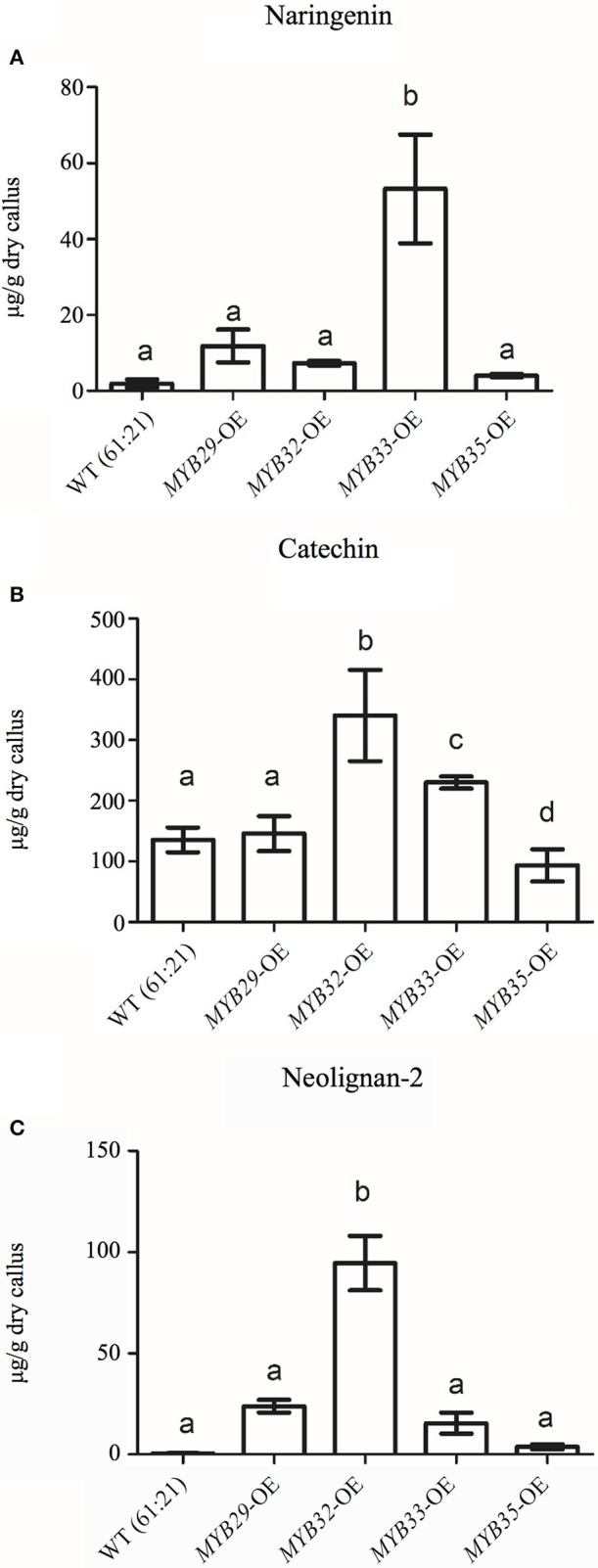
**Average naringenin (A)**, catechin **(B)**, and neolignan-2 **(C)** contents in μg/g of dry callus for WT (*p* < 0.05) and the transformant lines overexpressing *MYB29, MYB32, MYB33*, and *MYB35* (*n* = 3). Letters indicate statistical differences (*p* > 0.05) in a one-way ANOVA with Dunns post-test (*P* < 0.05).

The flavonoid, stilbene and neolignan profiles differed between lines overexpressing *PaMYB29, PaMYB32, Pa*MYB33, and *PaMYB35*, and WT material according to the PCA of these metabolite groups (Supplemental Material 9). The PCA revealed that the variation in flavonoids was mainly accounted for by differences in catechin and naringenin content between lines overexpressing different MYB genes (Supplemental Material 9A). Subsequent one-way ANOVA analyses verified significant differences in the levels these two metabolites between lines. Catechin, levels were higher in lines overexpressing *PaMYB32* and *PaMYB33*, compared to WT and to the lines overexpressing *Pa*MYB29 and *Pa*MYB35, the latter actually showing a significant lower catechin content than WT and lines overexpressing *Pa*MYB29 (Figure 7B). Naringenin levels were significantly elevated in lines overexpressing *Pa*MYB33 (Figure 7A), Stilbene profiles were not different between lines overexpressing different R2R3-MYB TFs but all lines exhibited stilbene profiles different from WT material (Supplemental Material 9C). Neolignan-2 (β-D-Xylopyranoside, 4-[1,3-dihydroxy-2-[2-hydroxy-4-(3-hydroxypropyl)-phenoxy]-propyl]-2-methoxyphenyl) explained 98% of the variation in neolignans in our dataset (Supplemental Material 9B). The subsequent one-way ANOVA analysis of neolignan-2 levels in the overexpression lines showed that lines overexpressing *Pa*MYB32 had significantly higher neolignan-2 levels compared to WT and the lines overexpressing *Pa*MYB29, *Pa*MYB33, and *Pa*MYB35 (Figure 7C).

## Discussion

Plants depend on (TFs) to respond and adapt to environmental changes. For this reason, plants possibly possess a higher TF diversity than other living organisms (Shiu et al., 2005). The amount of TFs varies between plant species, but higher plants generally have expanded TF gene families (Lang et al., 2010). Even though previous studies suggested that conifers have a lower number of TFs than angiosperms (Rigault et al., 2011; Canales et al., 2014), the publication of their genomes has shown that the TF number is similar but that TF families evolved differently in conifers, showing expansion or contraction of specific subgroups within TF families (Bedon et al., 2010; Nystedt et al., 2013). In this study we explored the specific diversity of a MYB-bHLH-WDR (MBW) complex in Norway spruce and compare our results with the present knowledge in conifers.

The WD40 protein is portrayed as the ubiquitous member of modular MBW complexes (Ramsay and Glover, 2005; Xu et al., 2015). In *Arabidopsis*, the WD40 member is represented by the stably expressed single-copy gene *AtTTG1*, which influences all traits associated with the MBW complex (Tominaga-Wada et al., 2011). Consistent with these reports, we find a single potential homolog of *AtTTG1* in Norway spruce, *PaWD40-1*, which is constitutively expressed in all tissues and in response to various types of abiotic stressors as expected of an *AtTTG1* Homolog. The predicted *Pa*WD40-1 protein sequence shows substantial identity and similarity to *At*TTG1, particularly in the C-terminal region, which has been shown to be important for the interaction between *At*TTG1 with the bHLH TF member of the complex, *At*TT8 (Matsui and Ohme-Takagi, 2010). Furthermore, consistent with the conservation of the predicted interaction domain, *Pa*WD40-1 interacts with the tested Norway spruce bHLH proteins included in the yeast two-hybrid assay. We could not detect direct physical interactions between *Pa*WD40-1 and any of the *Pa*MYB proteins and we do not preclude that the proteins may still interact *in vivo*. This also has been reported in *Arabidopsis* (Baudry et al., 2004), where direct interactions between TTG1 and TT2 homologs were not observed, and in strawberry (Schaart et al., 2013). Directionality appears to be relatively common in yeast two-hybrid assays involving members of MBW complexes and it has been attributed to partial misfolding possibly producing false negatives (Baudry et al., 2004; Schaart et al., 2013). Taken together, our data suggest that *PaWD40-1* is the *TTG1* homolog in Norway spruce.

R/B-like subgroup IIIf bHLH proteins participate in MBW complexes and some of these proteins in subgroup IIIf are involved in the control of pigmentation and flavonoid biosynthesis in angiosperms (Park et al., 2007; Park, 2012; Schwinn et al., 2014). Our study gives a first insight into this subgroup of bHLH TFs in conifers. The phylogenetic analyses of our bHLH candidates suggested that the three paralogous bHLH proteins are the closest homologs to *At*TT8, as the three bHLH candidates showed a shorter phylogenetic distance to *At*TT8 than to the other *Arabidopsis* bHLH subgroup IIIf members. Further analysis of the predicted amino acid sequences suggested that *PabHLH-1* and *PabHLH-2* encode functional bHLH proteins and that *PabHLH-3* is, most likely, a pseudogene. *Pa*bHLH-3 lacks large parts of the ID, AD and bHLH domains, which are essential for TT8 function (Pattanaik et al., 2008; Feller et al., 2011). Thus, we conclude that the Norway spruce genome contains two functional *AtTT8* homologs, *PabHLH-1* and *PabHLH-2*. However, the level of sequence divergence between the paralogs and the identification of orthologous sequences to both genes in the white spruce and loblolly pine genomes indicate that it is not a recent duplication but that it predates the divergence between *Picea* and *Pinus*, approximately 90–100 Mya (Lu et al., 2014). Even though *PabHLH-1* and *PabHLH-2* show similar expression patterns in most tissues and in response to abiotic stress, their protein interaction with subgroup 5 R2R3-MYB is largely dissimilar. *Pa*bHLH-2 interacts with the MYB proteins of the subgroup S5, *Pa*MYB29, *Pa*MYB31, and with *Pa*MYB32 and *Pa*MYB33, while *Pa*bHLH-1 only interacts with *Pa*MYB33 (Figure 5). Thus, the two paralogs appear to have diverged functionally since their separation.

In *Arabidopsis*, the R2R3-MYB TF subgroup 5 is represented by the single member *AtTT2* (Nesi et al., 2001), but in the legume model plant *Lotus japonicus* there are three members of this subgroup capable of restoring function in *tt2* mutants (Yoshida et al., 2008). When co-expressed with *LjTT8* and *LjTTG1*, the three *LjTT2*s show different activation of late biosynthetic gene transcription in the flavonoid biosynthesis pathway (Yoshida et al., 2010) and this variation in activation strength is associated with substitutions in the amino acid sequences of the *LjTT2*s. Previously, two different genes with similarity to *AtTT2* have been reported from the genus *Picea* (Xue et al., 2003; Arnerup, 2011), leading us to hypothesize that the conifer R2R3-MYB TF subgroup 5 also would contain multiple members with different functions. We isolated the cDNA sequence from seven Norway spruce R2R3-MYB TFs. Based on phylogenetic analysis, we suggest that six of these genes *PaMYB29, PaMYB30, PaMYB31, PaMYB32, PaMYB33*, and *PaMYB35* are members of, or highly similar to members of, subgroup 5 of the R2R3-MYB TF family. The phylogeny also indicated that the R2R3-MYB TFs have different evolutionary paths; *Pa*MYB29, *Pa*MYB30, and *Pa*MYB31 cluster together with *At*TT2 (Nesi et al., 2001) and its homologs in angiosperms (Pazares et al., 1987; Mellway et al., 2009; Terrier et al., 2009) forming the subgroup S5, while *Pa*MYB35 shows similarity to *Vv*MBPA1 and WPS-II SG MYBs of woody angiosperms (Bogs et al., 2007; Soler et al., 2015). However, *Pa*MYB32 and *Pa*MYB33 have no known homologs outside the gymnosperms, indicating that they represent a gymnosperm-specific subgroup of the R2R3-MYB TF family, which we named CS-I. Examples of R2R3-MYB family subgroup expansions in conifers compared to *Arabidopsis* have already been reported as it is the case for the expanded R2R3-MYB family subgroup 4, which was shown to be involved in the regulation of early flavonoid biosynthesis genes in conifers (Bedon et al., 2010; Bomal et al., 2014). Soler et al. (2015) reported that subgroup 5 is one of three R2R3–MYB TF subgroups, all controlling proanthocyanidin and anthocyanin pathways, which appear to be expanded in woody perennial angiosperms compared to herbaceous angiosperms. Thus, the formation of woody tissues may depend on a more refined regulation of these pathways, and that some of these subgroups may predate the separation of angiosperm gymnosperm lineages while others appear lineage specific.

Grotewold (2005) proposed a model to explain the functional divergence of recently duplicated regulatory genes and how that relates to metabolic diversity in plants. In that model, “the duplication of a regulatory gene for a metabolic pathway is followed by mutations that result in a TF with a partial loss of function” modulating the expression of some but not all genes in a pathway and allowing intermediates to accumulate. This sub-functionalization of the recently duplicated regulatory genes is then followed by its apparent neo-functionalization of metabolic pathways. Thus, under this model, the paralogous R2R3-MYB TFs identified in Norway spruce would regulate genes in the flavonoid pathway differently. To test this, we generated transgenic lines overexpressing *PaMYB29, PaMYB32, PaMYB33*, and *PaMYB35*. Overexpression of either of these TFs lead to higher expression levels of *PaLAR3* compared to wild type lines. *Pa*LAR3 catalyzes the last step in the flavonoid biosynthesis pathway to produce catechin and it is a key factor in resistance of Norway spruce to fungal pathogens (Hammerbacher et al., 2014; Nemesio-Gorriz et al., 2016). Interestingly, *PaMYB32*, the Norway spruce ortholog of *PmMBF1* (Xue et al., 2003), appeared to also specifically regulate *PaLAR3* as well as *PaLAR4*, while overexpression of its close paralog, *PaMYB33*, activated the expression of *PaPAL1* and *PaANR2, PaANR3, PaANR5, PaLAR1, PaLAR3*, and *PaLAR4* encoding enzymes in the late flavonoid biosynthesis. Together with their contrasting transcriptional responses to hormones and abiotic stress, our observations suggest that the gene duplication that gave rise to *PaMYB32* and *PaMYB33* was followed by a sub-functionalization of the paralogs, as predicted by the model on functional divergence of recently duplicated regulatory genes (Grotewold, 2005). Similar evolutionary changes have been observed for R2R3-MYB family TFs in several angiosperms (Dias et al., 2003; Chai et al., 2014; Zhao and Bartley, 2014).

The contrasting expression pattern of the gene pair *PaMYB32* and *PaMYB33* is the most obvious example, in this study, of distinct separation of tissue- or stress-dependent expression patterns between the R2R3–MYB TFs, but all studied genes show individual expression patterns. Such variation in expression patterns is consistent with the proposed modularity of the MBW complex where an exchange, or the tissue specificity, of the R2R3-MYB TFs would specify the regulation of individual traits (Dias et al., 2003; Baudry et al., 2004; Gonzalez, 2009; Xu et al., 2015) in combination with particular bHLH proteins (Ramsay and Glover, 2005). The gene expression profiles of the Norway spruce R2R3-MYB TFs and the expression patterns of late genes in flavonoid biosynthesis in the overexpression lines could indicate that the previously isolated genes *PaMYB29* and *PaMYB32* (Xue et al., 2003; Arnerup, 2011) control specific functions irrespective of tissue. The two genes were expressed at similar levels in most tested tissues, responded to abiotic and biotic stress, and *PaMYB29* and *PaMYB32* overexpressing lines showed an upregulation of specific genes in flavonoid biosynthesis. Interestingly enough, the gene expression patterns in the *PaMYB29* overexpression lines suggest that this particular TF may contribute to the induction of the genes *PaANR3* and *PaLAR3* in response to biotic stress. The more generalized late-biosynthesis-gene upregulation seen in *PaMYB33* and *PaMYB35* overexpression lines and the expression pattern of *PaMYB30, PaMYB31, PaMYB33*, and *PaMYB35*, which showed a higher degree of tissue specificity and more restricted responses to abiotic stress compared to *PaMYB29* and *PaMYB32*, suggests that these R2R3-MYB family TFs may perform their regulatory role in particular organs or distinct cell types. Such organ specificity is reported for *Arabidopsis*' subgroup 7 R2R3-MYB members (Stracke et al., 2007), for paralogous R2R3-MYB genes, *Pd*MYB2 and *Pd*MYB20, in poplar (Chai et al., 2014) and to some extent among conifer subgroup 4 R2R3-MYB family TFs (Bedon et al., 2007, 2010).

Previously, transcriptional profiling studies have shown that overexpression of conifer R2R3-MYBs acting on the shikimate and monolignol pathways can regulate specific sets of genes depending on the MYB that is overexpressed (Bedon et al., 2014). This regulation could potentially channel carbon flux into different directions leading to the accumulation of different metabolites, however such changes in metabolite patterns was not confirmed by metabolite profiling. In this study we attempted to correlate activation of genes in the flavonoid pathway with the soluble flavonoid, stilbene and lignan levels in the overexpression lines. The R2R3-MYB overexpressing lines showed higher expression levels of members of the *PaLAR* gene family compared to WT controls and overall this was reflected in their chemical profiles. All R2R3-MYB overexpressing lines, except lines overexpressing *PaMYB35*, had significantly higher catechin levels. This apparent incongruence between gene expression and metabolite accumulation in lines overexpressing *PaMYB35* could be an effect of substrate unavailability for particular enzymatic processes, due to the general activation of the late flavonoid biosynthesis pathway, leading to lower metabolite accumulation despite high levels of gene expression (Treutter, 2005). Furthermore, the possibility that the products made by the activated flavonoid biosynthesis pathway were laid down as insoluble lignin or proanthocyanidins affecting the profiles of soluble phenolics cannot be excluded. In fact, Bomal et al. (2008) observed reduced levels of soluble phenolics and an increased deposition of lignin and in *PtMYB1* and *PtMYB8* overexpressing white spruce lines. Despite the difficulties to find simple correlations of specific specialized metabolites with the overexpression of specific Norway spruce subgroup 5 R2R3-MYB TFs, the general impression is that several of the Norway spruce subgroup 5 R2R3-MYB TFs regulate the activity of the genes in the late flavonoid biosynthesis pathway and the subsequent accumulation flavonoids.

## Concluding discussion

Comparative analyses of gymnosperm and angiosperm TF gene families provided evidence of monophyletic gene family expansions that occurred in gymnosperms after the angiosperm–gymnosperm split (Guillet-Claude et al., 2004; Liu and Ekramoddoullah, 2004; Bedon et al., 2010) and illustrates the different evolutionary trajectories of the gene families in seed plants (Bedon et al., 2010). Our study on selected members of the MBW complex in Norway spruce further adds to this picture as we identified a *bHLH* gene pair orthologous to *AtTT8* and several members of the R2R3-MYB TF family subgroup 5 in the conifer, Norway spruce. Taken together, previous studies on conifer MBW members and this study indicate that conifer Subgroups 4 and 5 control early and late gene expression in the flavonoid pathway as well as stilbene and neolignan biosynthetic pathways. To fulfill these functions, it is likely that they form complexes with bHLH and WD40 proteins as suggested by Zhao et al. (2013). Testing interactions of *Pa*bHLH-1 and *Pa*bHLH-2 with members of R2R3-MYB subgroups 4 and 5 would shed light on the involvement of the bHLH paralogs in the control of the different biosynthetic modules. Furthermore, overexpression of members of R2R3-MYB under inducible promoters or silencing expression by RNA-interference might lead to a clearer elucidation of their functions.

## Author contributions

MN and ME conceived the study. MN isolated the candidate genes, performed the phylogenetic analyses, prepared the vectors for yeast and spruce transformation, extracted the RNA and performed the expression studies on plant tissues and cell lines. PB performed the protein interaction experiment and analyzed the data together with SM. JA performed the expression study to test biotic and abiotic stress on spruce plants. KD conducted the experiment to test abiotic stress on spruce cell lines. AH performed the chemical analysis of the cell lines. MN drafted the manuscript and coordinated the writing. PB, KD, AH, JS, SM, and ME contributed with advice on data analyses and advice during the process of writing the manuscript. MN wrote the final manuscript and all co-authors read and approved the final version of the manuscript.

### Conflict of interest statement

The authors declare that the research was conducted in the absence of any commercial or financial relationships that could be construed as a potential conflict of interest.
