# Supplementary material for: Identification of Norway Spruce MYB-bHLH-WDR Transcription Factor Complex Members Linked to Regulation of the Flavonoid Pathway
Source: Front Plant Sci. 2017 Mar 9;8:305. doi: 10.3389/fpls.2017.00305 (PMC5343035; doi:10.3389/fpls.2017.00305)
Supplement: Supplementary file 1 [file SupplementalMaterial1.pdf]

**OE-line**	**Expresion**
*Pa*MYB29-1	15.61
*Pa*MYB29-2	12.76
*Pa*MYB29-3	11.91
*Pa*MYB32-1	20.06
*Pa*MYB32-2	11.23
*Pa*MYB32-3	13.69
*Pa*MYB33-1	13.26
*Pa*MYB33-2	10.04
*Pa*MYB33-3	11.49
*Pa*MYB35-1	14.47
*Pa*MYB35-2	17.86
*Pa*MYB35-3	12.11

Supplementary material 1. Expression fold of the target genes in the transformant lines compared to WT lines.
