# Supplementary material for: Identification of Norway Spruce MYB-bHLH-WDR Transcription Factor Complex Members Linked to Regulation of the Flavonoid Pathway
Source: Front Plant Sci. 2017 Mar 9;8:305. doi: 10.3389/fpls.2017.00305 (PMC5343035; doi:10.3389/fpls.2017.00305)
Supplement: Supplementary file 2 [file SupplementalMaterial2.pdf]

**Figure 2**	
*Pa*MYB29	KU131218
*Pa*MYB30	KU131219
*Pa*MYB31	KU131220
*Pa*MYB32	KU131221
*Pa*MYB33	KU131222
*Pa*MYB34	KU131223
*Pa*MYB35	KU131224
*Pg*MYB10	ABQ51221
*Pg*MYB13	ABQ51226
*Pg*MYB14	ABQ51229
*Pg*MYB15	ACN12953
*Pg*MYB16	ACN12954
*Pg*MYB17	ACN12958
*Pg*MYB18	ACN12959
*Pg*MYB19	ACN12955
*Pg*MYB20	ACN12956
*Pg*MYB29	ACN12957
*Pg*MYB30	BT116752*
*Pg*MYB31	BT118097*
*Pg*MYB32	BT119291*
*Pg*MYB33	BT116479*
*Pg*MYB35	BT109791*
*Pm*MBF1	AAA82943
*Pt*MYB5	ACN12960
*Pt*MYB10	ACN12961
*Pt*MYB13	ACN12962
*Pt*MYB14	ABD60279
*Pt*MYB16	FJ469927
*Pt*MYB21	ACN12964
*Pt*MYB134	ACR83705
*Vv*MYBPA1	NP_001268160
*Vv*MYBPA2	ACK56131
*Zm*C1	NP_001147547
*At*TT2	NP_198405
*At*MYB3	NP_564176
*At*MYB32	NP_195225

**Figure 1**	
*Pa*bHLH-1	KU131226
*Pa*bHLH-2	KU131227
*Pa*bHLH-3	KU131228
*At*TT8	NP_192720
*At*MYC1	AEG74527
*At*GL3	NP_680372
*At*EGL3	NP_001185302
*At*bHLH4	AAL55711
*At*bHLH5	AAL55712
*At*bHLH28	AAL55721
*At*RAP-1	Q39204
*At*MYC7E	AAM53310
*At*bHLH14	CAD58596
*At*bHLH3	AAL55710

**Other**	
PaWDR40-1	KU131225

Supplemental material 2. EMBL/GenBank accession numbers for Figure 1 and Figure 2. * indicates cDNA accession that was used to predict a protein sequence.
