# Supplementary material for: Identification of Norway Spruce MYB-bHLH-WDR Transcription Factor Complex Members Linked to Regulation of the Flavonoid Pathway
Source: Front Plant Sci. 2017 Mar 9;8:305. doi: 10.3389/fpls.2017.00305 (PMC5343035; doi:10.3389/fpls.2017.00305)
Supplement: Supplementary file 3 [file SupplementalMaterial3.pdf]

AttB primers	AttB1	AttB2
PaMYB29	GGGGACAAGTTTGTACAAAAAAGCAGGCTTGTAGCCTCCTGCTGGTTGAGTGTTTG	GGGGACCACTTTGTACAAGAAAGCTGGGTGGAACAAGGATAGAAAGCTCGCCCAAAT
PaMYB30	GGGGACAAGTTTGTACAAAAAAGCAGGCTCCGACGAAGAAATTCCAAGATGG	GGGGACCACTTTGTACAAGAAAGCTGGGTCGGAATATTATCCAGGGATTACG
PaMYB31	GGGGACAAGTTTGTACAAAAAAGCAGGCTCCACGAAGAAAGTCCAAGATGG	GGGGACCACTTTGTACAAGAAAGCTGGGTCAAACATCATACCACATCTGG
PaMYB32	GGGGACAAGTTTGTACAAAAAAGCAGGCTCCAGTCGTTCCAGTAGAGTAGAGATGG	GGGGACCACTTTGTACAAGAAAGCTGGGTCGGGGAAACAGAAAAAGACTAACC
PaMYB33	GGGGACAAGTTTGTACAAAAAAGCAGGCTCCATCCTGCATTGTATTGGAAGTAG	GGGGACCACTTTGTACAAGAAAGCTGGGTCCTGTCTGGAAAGACCTTTACATTAG
PaMYB34	GGGGACAAGTTTGTACAAAAAAGCAGGCTCCTTCCTCAGCATCTCACACAGTATT	GGGGACCACTTTGTACAAGAAAGCTGGGTCAGGGAGTGGGAAGAAGAATCTATG
PaMYB35	GGGGACAAGTTTGTACAAAAAAGCAGGCTCCTAGTTAGGAATCGAGTGGAAATGG	GGGGACCACTTTGTACAAGAAAGCTGGGTCGATACTACCAGGGCTGAATCCTCT
PabHLH-1	GGGGACAAGTTTGTACAAAAAAGCAGGCTGGTATCTTTGGAGCTGGGGCAGACTTT	GGGGACCACTTTGTACAAGAAAGCTGGGTTCTCTTTGGCACTGGACGTCTCCATAA
PabHLH-2	GGGGACAAGTTTGTACAAAAAAGCAGGCTCCCTTATGTGCCTGAAGGCTATG	GGGGACCACTTTGTACAAGAAAGCTGGGTCTCTATCAGTTCAGCATTTGGAG
PabHLH-3	GGGGACAAGTTTGTACAAAAAAGCAGGCTCCTTGAAATCTGAAACCATGGC	GGGGACCACTTTGTACAAGAAAGCTGGGTCTCACCCATTGACAGAAAAGAG
PaWD40-1	GGGGACAAGTTTGTACAAAAAAGCAGGCTCCGCAGGGCACTGGTATTTAGAA	GGGGACAAGTTTGTACAAAAAAGCAGGCTCCCAAATGAGAGACTGGCAAAAGTT

qPCR primers	Forward	Reverse
PaMYB29	TTGCGTTGGCTGAACTATCTT	CAAGAAGACGATGTAATCTGACG
PaMYB30	GCAATATGGAGCGCCTTAAC	CGGGGCTTGATTTCTGTAAA
PaMYB31	CTCCATCCAATTGTTCAGCA	TCTCGCTGCATAATGCTTTG
PaMYB32	CGATTCAACCGAAGTCCAAT	CACAGCTAGCAGCAGAGTCG
PaMYB33	TGCCATTGGAATGAGAAACA	TCGGCGGATTCTTCATCTAC
PaMYB34	GGCCGAACTGACAATGAAAT	GACGCTTCAATGTTTGAGCA
PaMYB35	AAAGGAATCGACCCCAAAAC	CTGTCATACCGTGCTCGAAA
PabHLH-1	TTCAGAAAACGCATCACGAG	CGTGGGCATTGTCTGAGTTA
PabHLH-2	GGTCTAGTCCAACGCACCAT	CATGCCCACTTGATCCTTTT
PabHLH-3	GTTTACCAGGTCGGGCACTA	AGGTACCTGAATGCCAGCAC
PaWD40-1	GAATTGGGACATCGAGCATT	CTACCTCACCCCAGGCAATA
EF4a	AGTAAGCCCGTGGAGGATTC	AGTCAGCCAGTCAACCTTTC
PAL1	GGCAGATCATTTGGGTGATC	TAAAGTTCCATTTTCAACTATAGGACTAAT
CHS	CCGCCTCTCAAATAAATCGTATTAGT	ATCAATTATTTGGGTTTCAGTTCTG
ANR2	ATGGAGAACCCCTCTGCATAC	TCAAATTGGGTTGTCACATTGT
ANR3	ATGGAGAACCCCTCTGCACAG	TCAAACTCGGTTGGGACATTGTA
ANR5	TATGGAGAACCCTTCTACAGA	TCAAATTGGGTAGGTACG
LAR1	GAACTGGCAGCCATATGGGAGACC	CACCTCTCAGACGAAGTAC
LAR2	ACAAGAACTTTTGCATTTAGCCG	GAAATCTCTGGATATAGTTGTGAC
LAR3	CGGACATTGTGACACGAAAC	CGGAGTTTATACCCGTTCCA
LAR4	TTAGGATAGCTGAGGCGAACTT	TGTATTTCAGGTCGGGATAGAGCT

Supplemental material 3. Primers used in this study.
