# Supplementary figures and images for: Identification of Norway Spruce MYB-bHLH-WDR Transcription Factor Complex Members Linked to Regulation of the Flavonoid Pathway
Source: Front Plant Sci. 2017 Mar 9;8:305. doi: 10.3389/fpls.2017.00305 (PMC5343035; doi:10.3389/fpls.2017.00305)

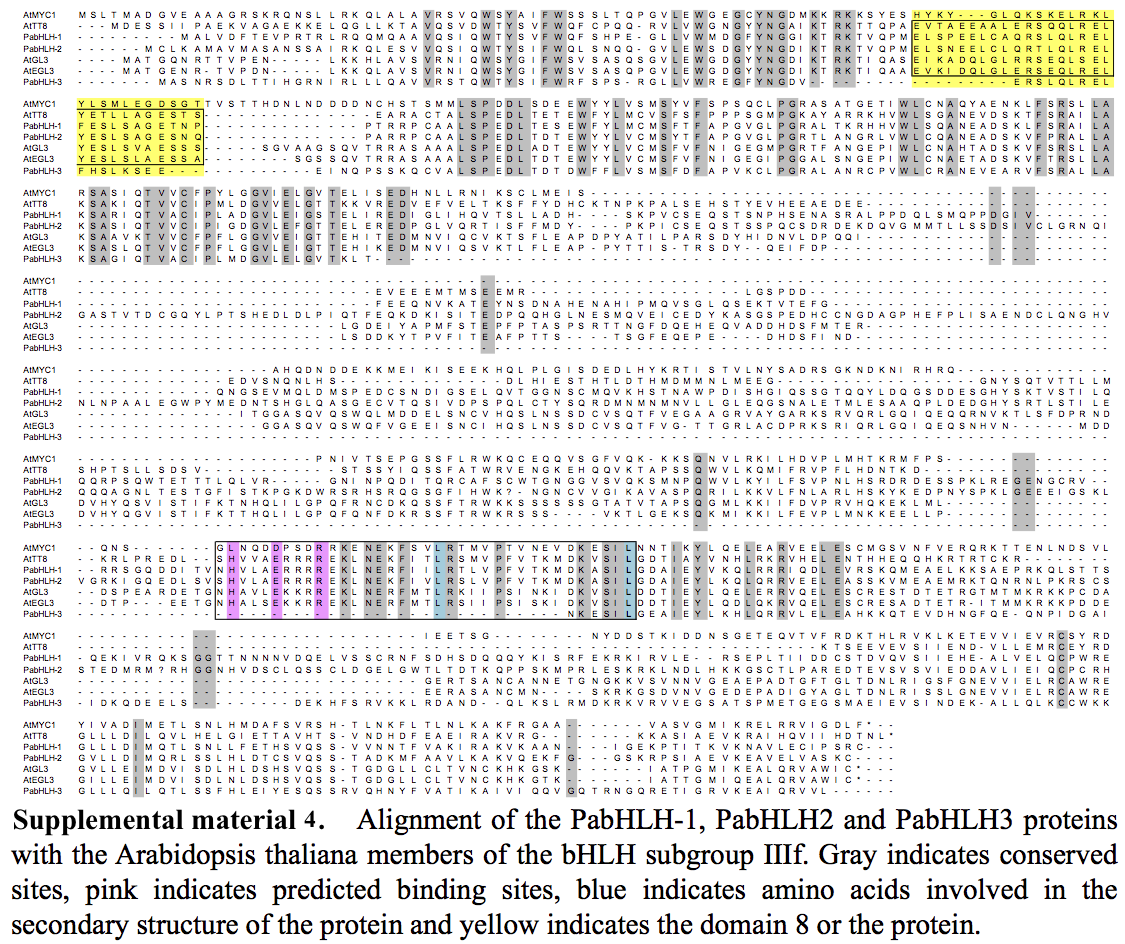

Supplement: Supplementary file 4 [file SupplementalMaterial4.tiff]

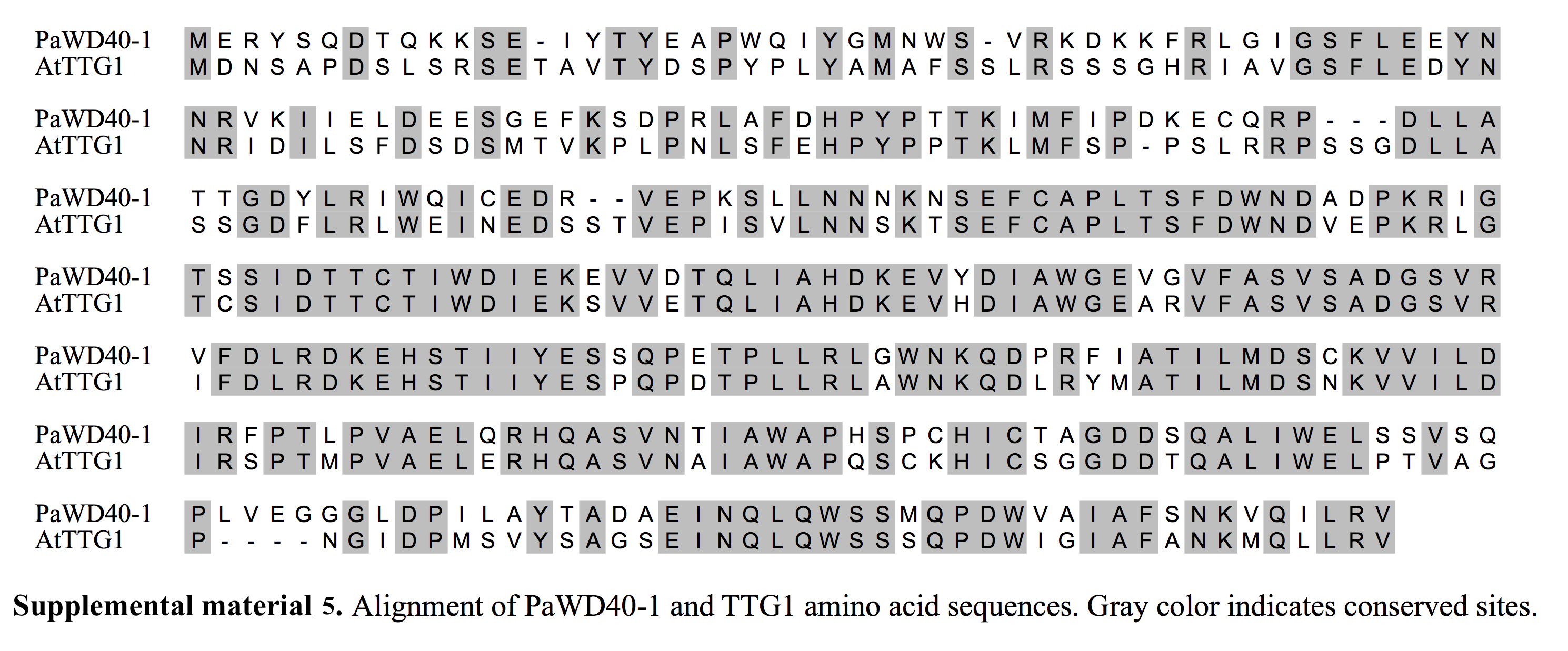

Supplement: Supplementary file 5 [file SupplementalMaterial5.tiff]

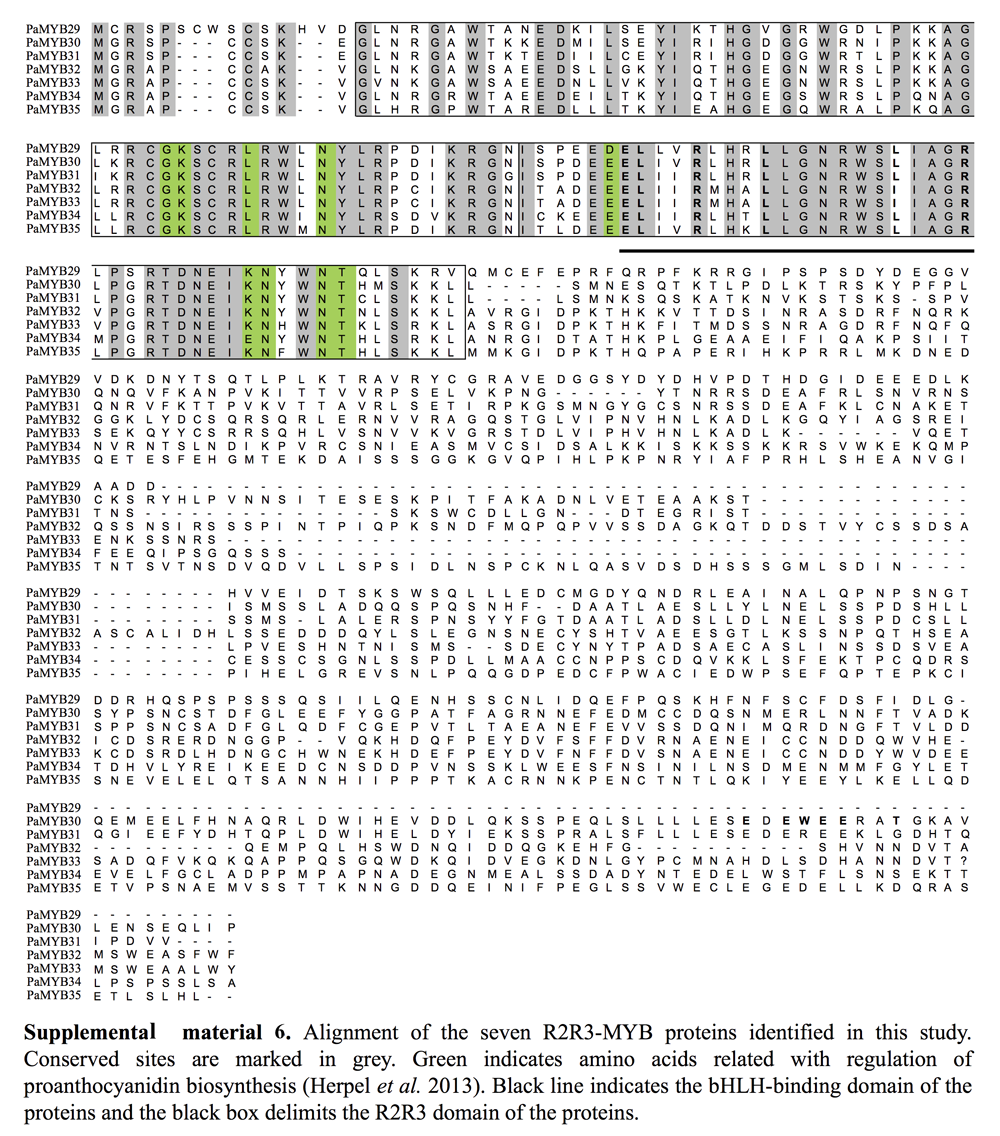

Supplement: Supplementary file 6 [file SupplementalMaterial6.tiff]

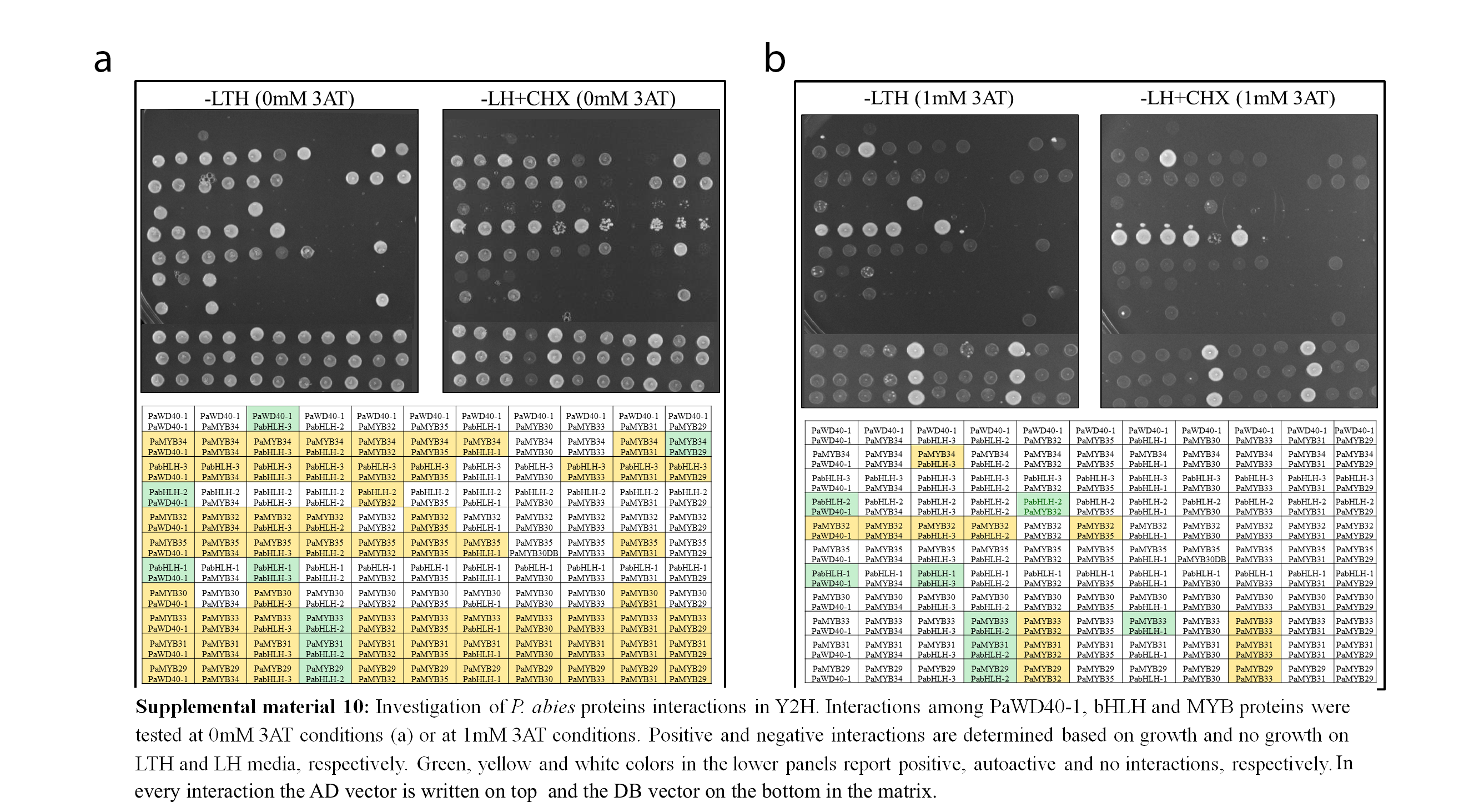

Supplement: Supplementary file 10 [file SupplementalMaterial10.tiff]
