# Supplementary material for: Identification of Norway Spruce MYB-bHLH-WDR Transcription Factor Complex Members Linked to Regulation of the Flavonoid Pathway
Source: Front Plant Sci. 2017 Mar 9;8:305. doi: 10.3389/fpls.2017.00305 (PMC5343035; doi:10.3389/fpls.2017.00305)
Supplement: Supplementary file 7 [file SupplementalMaterial7.pdf]

Gene	*Picea glauca* genome	*Picea glauca* GenBank	*Pinus taeda* genome	*Picea abies* genome	*Picea abies* GenBank	*Picea sitchensis* GenBank	*Picea mariana* GenBank
PaMYB29	Pg/01r141201s2042381	BT118801.1		MA_10069323	JF810440.1		
PaMYB30	Pg/01r141201s2438315	BT116752.1	tscaffold3501	MA_8147			
PaMYB31	Pg/01r141201s2588126	BT118097.1		MA_323706		EF084239.1	
PaMYB32	Pg/01r141201s2521057	BT119291.1	scaffold712432.1	MA_130918			U39448.1
PaMYB33	Pg/01r141201s2552601	BT116479.1	tscaffold1375	MA_140579		BT123774.1	AY166640.1
PaMYB34	Pg/01r141201s1890156		tscaffold2248	MA_93127			AY166636.1
PaMYB35	Pg/01r141201s1951792	BT109791.1	scaffold156658.1	MA_53373			
PaWD40-1	Pg/01r141201s2988389	BT110191.1	scaffold563497	MA_6946339		EF676613.1	
PabHLH-1	Pg/01r141201s2430524	BT114491.1	scaffold312531.1	MA_96585			
PabHLH-2	Pg/01r141201s2072888		scaffold481050	MA_17689		EF678027.1	
PabHLH-3	Pg/01r141201s1883853	BT109812.1		MA_8255			

Supplemental Material 7. Genome and GenBank references of the genes in this study in conifer species.
