# Supplementary material for: Identification of Norway Spruce MYB-bHLH-WDR Transcription Factor Complex Members Linked to Regulation of the Flavonoid Pathway
Source: Front Plant Sci. 2017 Mar 9;8:305. doi: 10.3389/fpls.2017.00305 (PMC5343035; doi:10.3389/fpls.2017.00305)
Supplement: Supplementary file 8 [file SupplementalMaterial8.pdf]

	Wounding 3dpi	Inoculation 3 dpi	Wounding 7dpi	Inoculation 7 dpi
PAL1	27.7 ±18.7*	104.0 ± 31.8*	17.9 ± 1.8*	120.8 ± 58.2*
ANR2	2.3 ± 1.3*	22.8 ± 18.2*	6.2 ± 3.7*	25.8 ± 10.4*
ANR3	9.2 ± 7.7*	13.9 ± 15.3*	6.1 ± 4.4*	10.9 ± 8.0*
ANR5	0.8 ± 0.3	0.9 ± 0.4	0.8 ± 0.2	1.4 ± 0.3
LAR3	2.5 ± 2.1	4.0 ± 2.1*	1.4 ± 0.6	3.3 ± 1.0*
LAR4	9.7 ± 3.7*	19.4 ± 5.8*	12.8 ± 3.0*	28.5 ± 2.7*
PaMYB29	22.5 ± 10.9*	47.5 ± 9.5*	23.2 ± 3.6*	99.8 ± 17.0*
PaMYB32	0.9 ± 0.1	1.1 ± 0.1	1.5 ± 0.6	2.0 ± 0.1*

Supplemental material 8. Expression level fold-change in Norway spruce bark wounded or inoculated with *H. parviporum* compared to untreated plant material. * indicate significant regulation of the gene compared to untreated control * ($p<0.05$, one/way ANOVA).
