# Supplementary material for: Identification of Norway Spruce MYB-bHLH-WDR Transcription Factor Complex Members Linked to Regulation of the Flavonoid Pathway
Source: Front Plant Sci. 2017 Mar 9;8:305. doi: 10.3389/fpls.2017.00305 (PMC5343035; doi:10.3389/fpls.2017.00305)
Supplement: Supplementary file 9 [file SupplementalMaterial9.pdf]

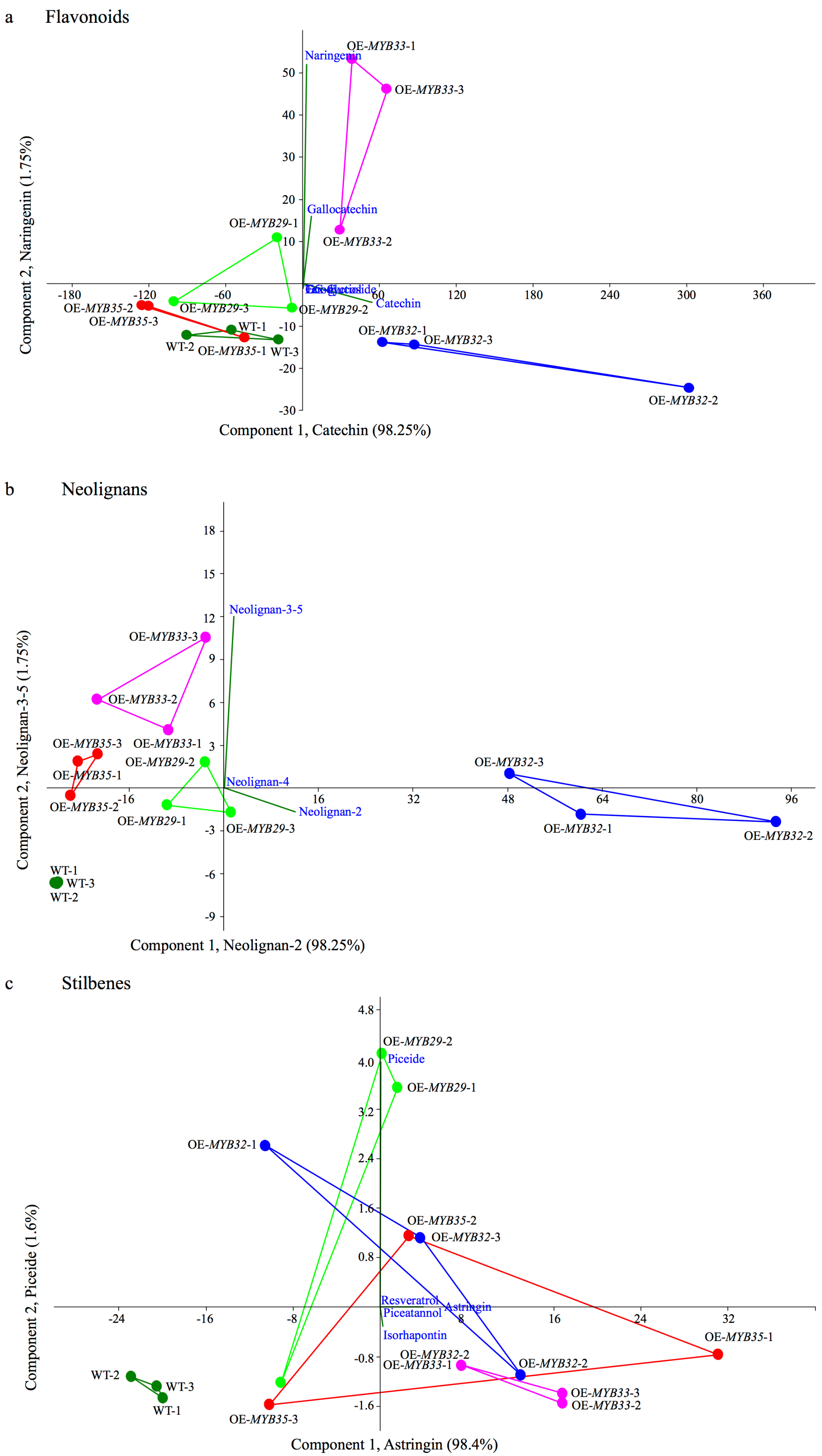

Supplemental material 9. Principal component analysis for flavonoids (a), neolignans (b) and stilbenes (c) in WT (dark green) and transformant lines overexpressing *MYB29* (light green), *MYB32* (blue), *MYB33* (pink) and *MYB35* (red).
